# The genome sequence of the Figwort Cheilosia, *Cheilosia variabilis *(Panzer, 1798)

**DOI:** 10.12688/wellcomeopenres.19851.1

**Published:** 2023-08-30

**Authors:** Liam M. Crowley, Olga Sivell, Michael Ashworth, Oliver Poole, University of Oxford and Wytham Woods Genome Acquisition Lab, Natural History Museum Genome Acquisition Lab, Darwin Tree of Life Barcoding collective, Wellcome Sanger Institute Tree of Life programme, Wellcome Sanger Institute Scientific Operations: DNA Pipelines collective, Tree of Life Core Informatics collective, Darwin Tree of Life Consortium

**Affiliations:** 1 University of Oxford, Oxford, England, UK; 2 Natural History Museum, London, England, UK; 3 Independent researcher, Yeovil, England, UK; 4 University of Exeter, Penryn, England, UK

**Keywords:** Cheilosia variabilis, Figwort Cheilosia, genome sequence, chromosomal, Diptera

## Abstract

We present a genome assembly from an individual male *Cheilosia variabilis* (the Figwort Cheilosia; Arthropoda; Insecta; Diptera; Syrphidae). The genome sequence is 414.7 megabases in span. Most of the assembly is scaffolded into 7 chromosomal pseudomolecules, including the X and Y sex chromosomes. The mitochondrial genome has also been assembled and is 16.77 kilobases in length.

## Species taxonomy

Eukaryota; Metazoa; Eumetazoa; Bilateria; Protostomia; Ecdysozoa; Panarthropoda; Arthropoda; Mandibulata; Pancrustacea; Hexapoda; Insecta; Dicondylia; Pterygota; Neoptera; Endopterygota; Diptera; Brachycera; Muscomorpha; Eremoneura; Cyclorrhapha; Aschiza; Syrphoidea; Syrphidae; Eristalinae; Rhingiini; *Cheilosi*a; *Cheilosia variabilis* (Panzer, 1798) (NCBI:txid273447).

## Background

*Cheilosia* is the most speciose hoverfly genus in Europe, however, because its species are mostly black and lack any obvious defining features, they are notoriously difficult to identify and often overlooked as hoverflies ( Vujic *et al.*, 2013). Members of the Cheilosiini tribe share a defined margin between the eye and the face called a ‘zygoma’. *C. variabilis* belongs to a small group of Cheilosia that possess distinctive uptstanding hairs between the zygoma and the central facial knob ( Ball & Morris, 2013; van Veen, 2010).

The Figwort Cheilosia, *Cheilosia variabilis*, is a large, black hoverfly with particularly long wings (wing length 7.75–10.25 mm) common throughout the Palearctic realm ( Ball & Morris, 2013). This species is frequently observed along woodland rides in deciduous forests, feeding on white umbellifers and settling on foliage with its wings held in a characteristic delta shape posture ( Speight, 2017; van Veen, 2010). *C. variabilis* are bivoltine, producing two broods of offspring per year during their flight period of April to September ( Speight, 2017). The larvae are phytophagous and bore into the rhizomes of the Common Figwort, *Scrophularia nodosa*, and the stalks of the Water Figwort, *S. auriculata*, hence its common name ( Coe, 1953; Dušek, 1962; Rotheray, 1990; Rotheray & Gilbert, 1999).

The distribution of the Figwort Cheilosia spans from Ireland to Western Siberia and Iran, and from Fennoscandia to Morocco, and it is also widespread across the United Kingdom ( Khaganinia & Kazerani, 2014). This species’ range and abundance across the Balkan Peninsula is predicted to decrease under current climate projections, highlighting their dependence on the cooler, more humid habitats found at the lower elevations of this vast mountainous region ( Radenković *et al.*, 2017). It is anticipated that this novel high-quality sequenced genome of *Cheilosia variabilis*, generated as part of the Darwin Tree of Life project, will help progress an understanding into this species’ biology and ecology.

## Genome sequence report

**Figure 1.**
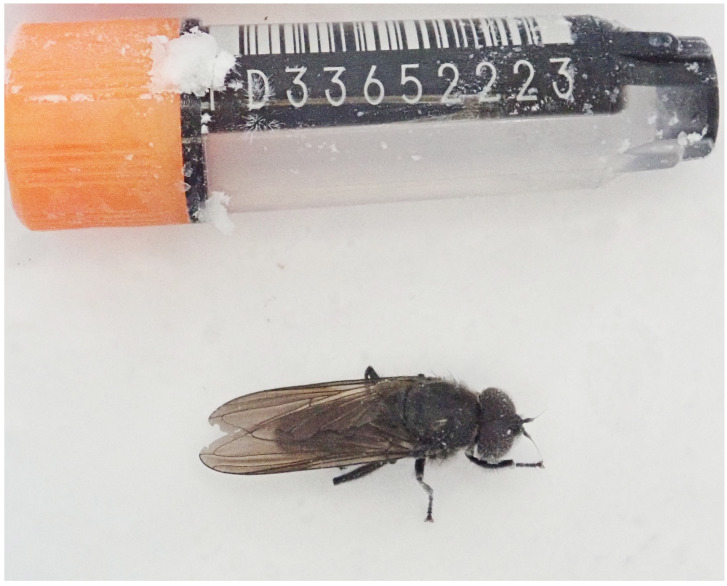
Photograph of the *Cheilosia variabilis* (idCheVari2) specimen used for genome sequencing.

The genome was sequenced from one male *Cheilosia variabilis* ( Figure 1) collected from Wytham Woods, Oxfordshire, UK (51.77, –1.33). A total of 63-fold coverage in Pacific Biosciences single-molecule HiFi long reads was generated. Primary assembly contigs were scaffolded with chromosome conformation Hi-C data. Manual assembly curation corrected 26 missing joins or misjoins and removed one haplotypic duplication, reducing the scaffold number by 66.67%, and increasing the scaffold N50 by 7.12%.

**Figure 2.**
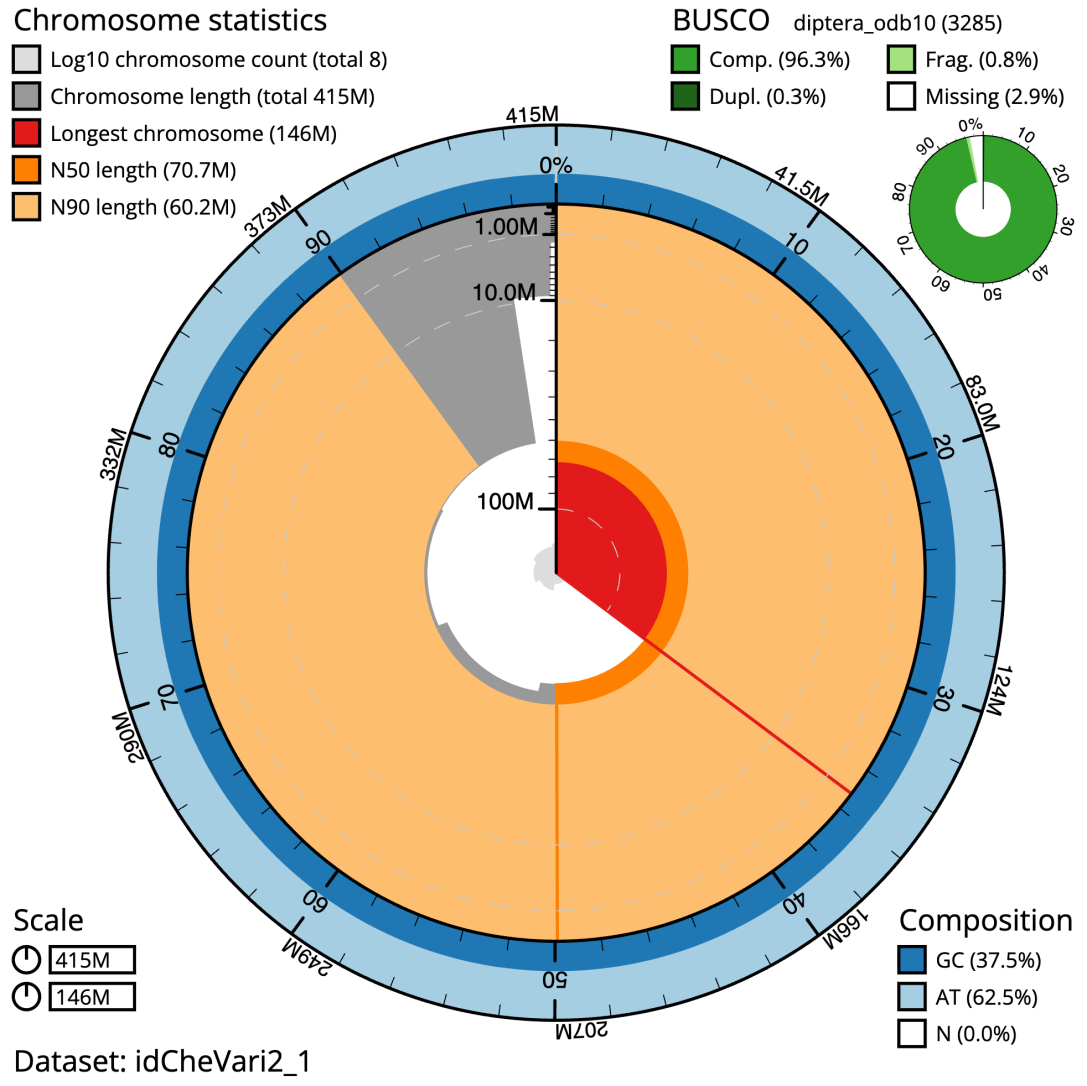
Genome assembly of *Cheilosia variabilis*, idCheVari2.1: metrics. The BlobToolKit Snailplot shows N50 metrics and BUSCO gene completeness. The main plot is divided into 1,000 size-ordered bins around the circumference with each bin representing 0.1% of the 414,757,870 bp assembly. The distribution of scaffold lengths is shown in dark grey with the plot radius scaled to the longest scaffold present in the assembly (146,055,565 bp, shown in red). Orange and pale-orange arcs show the N50 and N90 scaffold lengths (70,713,320 and 60,228,328 bp), respectively. The pale grey spiral shows the cumulative scaffold count on a log scale with white scale lines showing successive orders of magnitude. The blue and pale-blue area around the outside of the plot shows the distribution of GC, AT and N percentages in the same bins as the inner plot. A summary of complete, fragmented, duplicated and missing BUSCO genes in the diptera_odb10 set is shown in the top right. An interactive version of this figure is available at https://blobtoolkit.genomehubs.org/view/idCheVari2.1/dataset/idCheVari2_1/snail.

The final assembly has a total length of 414.7 Mb in 7 sequence scaffolds with a scaffold N50 of 70.7 Mb ( Table 1). Most (99.99%) of the assembly sequence was assigned to 7 chromosomal-level scaffolds, 5 autosomes and the X and Y sex chromosomes. Chromosome-scale scaffolds confirmed by the Hi-C data are named in order of size ( Figure 2– Figure 5; Table 2). The X and Y chromosomes were identified by read coverage. While not fully phased, the assembly deposited is of one haplotype. Contigs corresponding to the second haplotype have also been deposited. The mitochondrial genome was also assembled and can be found as a contig within the multifasta file of the genome submission.

**Table 1.** Genome data for *Cheilosia variabilis*, idCheVari2.1.

Project accession data		
Assembly identifier	idCheVari2.1	
Species	*Cheilosia variabilis*	
Specimen	idCheVari2	
NCBI taxonomy ID	273447	
BioProject	PRJEB59374	
BioSample ID	SAMEA110451622	
Isolate information	idCheVari2, male: thorax (DNA sequencing) idCheVari1: head and thorax (Hi-C scaffolding)	
Assembly metrics *		*Benchmark*
Consensus quality (QV)	68.9	*≥ 50*
*k*-mer completeness	100%	*≥ 95%*
BUSCO **	C:96.3%[S:96.0%,D:0.3%], F:0.8%,M:2.9%,n:3,285	*C ≥ 95%*
Percentage of assembly mapped to chromosomes	99.99%	*≥ 95%*
Sex chromosomes	X and Y chromosomes	*localised homologous pairs*
Organelles	Mitochondrial genome assembled	*complete single alleles*
Raw data accessions		
PacificBiosciences SEQUEL II	ERR10841318	
Hi-C Illumina	ERR10851511	
Genome assembly		
Assembly accession	GCA_951230905.1	
*Accession of alternate haplotype*	GCA_951230875.1	
Span (Mb)	414.7	
Number of contigs	102	
Contig N50 length (Mb)	7.9	
Number of scaffolds	7	
Scaffold N50 length (Mb)	70.7	
Longest scaffold (Mb)	146.1	

* Assembly metric benchmarks are adapted from column VGP-2020 of “Table 1: Proposed standards and metrics for defining genome assembly quality” from ( Rhie *et al.*, 2021). ** BUSCO scores based on the diptera_odb10 BUSCO set using v5.3.2. C = complete [S = single copy, D = duplicated], F = fragmented, M = missing, n = number of orthologues in comparison. A full set of BUSCO scores is available at https://blobtoolkit.genomehubs.org/view/idCheVari2.1/dataset/idCheVari2_1/busco.

**Figure 3.**
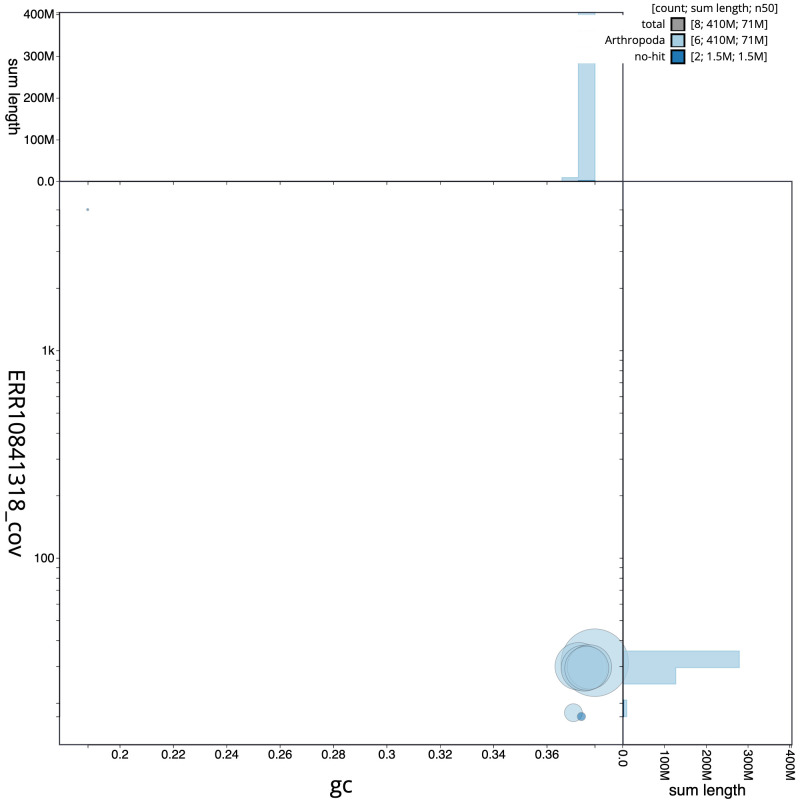
Genome assembly of *Cheilosia variabilis*, idCheVari2.1: BlobToolKit GC-coverage plot. Scaffolds are coloured by phylum. Circles are sized in proportion to scaffold length. Histograms show the distribution of scaffold length sum along each axis. An interactive version of this figure is available at https://blobtoolkit.genomehubs.org/view/idCheVari2.1/dataset/idCheVari2_1/blob.

**Figure 4.**
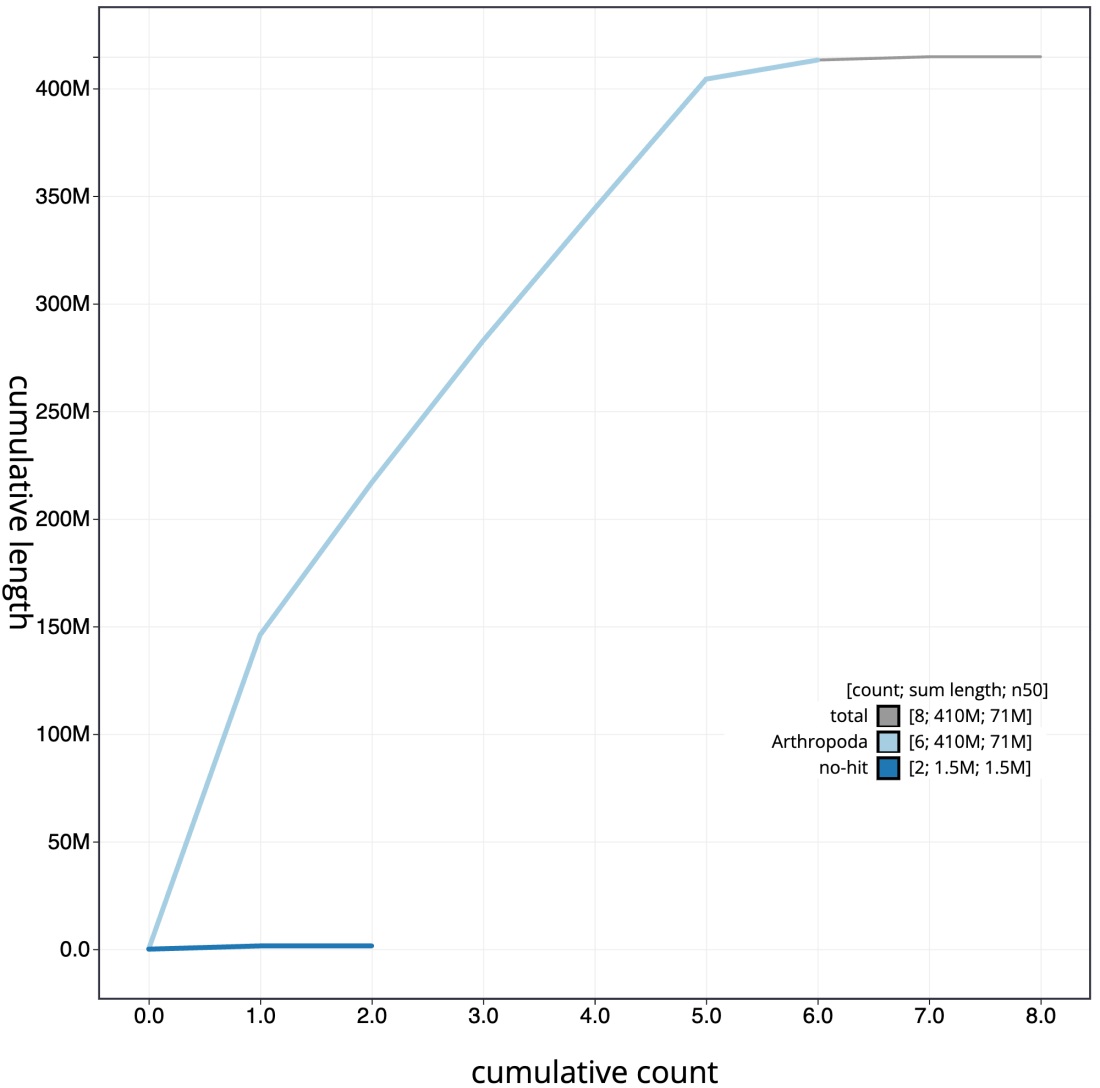
Genome assembly of *Cheilosia variabilis*, idCheVari2.1: BlobToolKit cumulative sequence plot. The grey line shows cumulative length for all scaffolds. Coloured lines show cumulative lengths of scaffolds assigned to each phylum using the buscogenes taxrule. An interactive version of this figure is available at https://blobtoolkit.genomehubs.org/view/idCheVari2.1/dataset/idCheVari2_1/cumulative.

**Figure 5.**
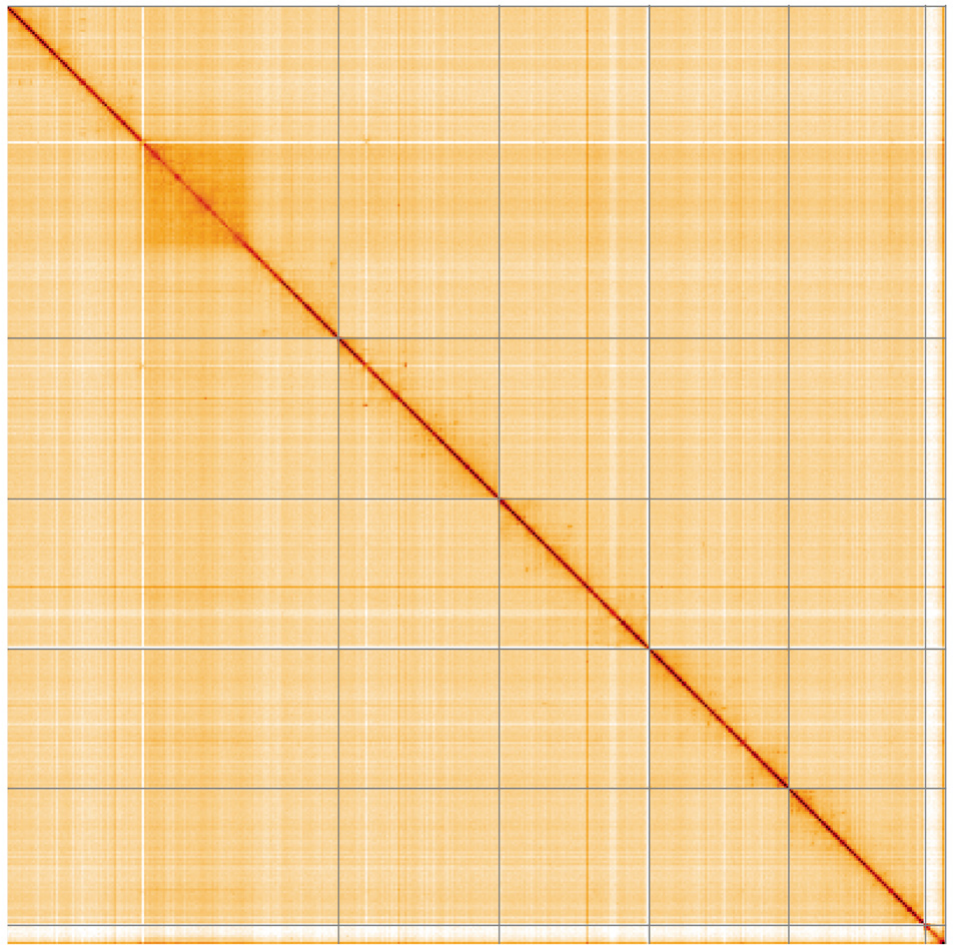
Genome assembly of *Cheilosia variabilis*, idCheVari2.1: Hi-C contact map of the idCheVari2.1 assembly, visualised using HiGlass. Chromosomes are shown in order of size from left to right and top to bottom. An interactive version of this figure may be viewed at https://genome-note-higlass.tol.sanger.ac.uk/l/?d=JLVp73kbRgqOs5W3nRNJjw.

**Table 2.** Chromosomal pseudomolecules in the genome assembly of *Cheilosia variabilis*, idCheVari2.

INSDC accession	Chromosome	Length (Mb)	GC%
OX579676.1	1	146.06	38.0
OX579677.1	2	70.71	37.0
OX579678.1	3	66.01	37.5
OX579679.1	4	61.34	37.5
OX579680.1	5	60.23	37.5
OX579681.1	X	8.88	37.0
OX579682.1	Y	1.51	37.5
OX579683.1	MT	0.02	19.0

The estimated Quality Value (QV) of the final assembly is 68.9 with *k*-mer completeness of 100%, and the assembly has a BUSCO v5.3.2 completeness of 96.3% (single = 96.0%, duplicated = 0.3%), using the diptera_odb10 reference set ( *n* = 3,285).

Metadata for specimens, spectral estimates, sequencing runs, contaminants and pre-curation assembly statistics can be found at https://links.tol.sanger.ac.uk/species/273447.

## Methods

### Sample acquisition and nucleic acid extraction

A male *Cheilosia variabilis* (specimen ID Ox002181, individual idCheVari2) was collected from Wytham Woods, Oxfordshire, UK (latitude 51.77, longitude –1.33) on 2022-05-19 by netting. The specimen was collected and identified by Liam Crowley (University of Oxford). The specimen used to generate Hi-C data (specimen ID NHMUK014452799, ToLID idCheVari1) was collected from the Eden Project, Cornwall (latitude 50.36, longitude –4.74) on 2021-06-28. This specimen was collected by Olga Sivell (Natural History Museum) and identified by Michael Ashworth (independent researcher). Both specimens were preserved on dry ice.

The idCheVari2 sample was prepared for DNA extraction at the Tree of Life laboratory, Wellcome Sanger Institute (WSI). The sample was weighed and dissected on dry ice. Thorax tissue was disrupted using a Nippi Powermasher fitted with a BioMasher pestle. DNA was extracted at the WSI Scientific Operations core using the Qiagen MagAttract HMW DNA kit, according to the manufacturer’s instructions.

### Sequencing

Pacific Biosciences HiFi circular consensus DNA sequencing libraries were constructed according to the manufacturers’ instructions. DNA sequencing was performed by the Scientific Operations core at the WSI on a Pacific Biosciences SEQUEL II (HiFi) instrument. Hi-C data were also generated from head and thorax tissue of idCheVari1 using the Arima2 kit and sequenced on the Illumina NovaSeq 6000 instrument.

### Genome assembly, curation and evaluation

Assembly was carried out with Hifiasm ( Cheng *et al.*, 2021) and haplotypic duplication was identified and removed with purge_dups ( Guan *et al.*, 2020). The assembly was then scaffolded with Hi-C data ( Rao *et al.*, 2014) using YaHS ( Zhou *et al.*, 2023). The assembly was checked for contamination and corrected as described previously ( Howe *et al.*, 2021). Manual curation was performed using HiGlass ( Kerpedjiev *et al.*, 2018) and Pretext ( Harry, 2022). The mitochondrial genome was assembled using MitoHiFi ( Uliano-Silva *et al.*, 2023), which runs MitoFinder ( Allio *et al.*, 2020) or MITOS ( Bernt *et al.*, 2013) and uses these annotations to select the final mitochondrial contig and to ensure the general quality of the sequence.

A Hi-C map for the final assembly was produced using bwa-mem2 ( Vasimuddin *et al.*, 2019) in the Cooler file format ( Abdennur & Mirny, 2020). To assess the assembly metrics, the *k*-mer completeness and QV consensus quality values were calculated in Merqury ( Rhie *et al.*, 2020). This work was done using Nextflow ( Di Tommaso *et al.*, 2017) DSL2 pipelines “sanger-tol/readmapping” ( Surana *et al.*, 2023a) and “sanger-tol/genomenote” ( Surana *et al.*, 2023b). The genome was analysed within the BlobToolKit environment ( Challis *et al.*, 2020) and BUSCO scores ( Manni *et al.*, 2021; Simão *et al.*, 2015) were calculated.

Table 3 contains a list of relevant software tool versions and sources.

**Table 3.** Software tools: versions and sources.

Software tool	Version	Source
BlobToolKit	4.1.7	https://github.com/blobtoolkit/blobtoolkit
BUSCO	5.3.2	https://gitlab.com/ezlab/busco
Hifiasm	0.16.1-r375	https://github.com/chhylp123/hifiasm
HiGlass	1.11.6	https://github.com/higlass/higlass
Merqury	MerquryFK	https://github.com/thegenemyers/MERQURY.FK
MitoHiFi	2	https://github.com/marcelauliano/MitoHiFi
PretextView	0.2	https://github.com/wtsi-hpag/PretextView
purge_dups	1.2.3	https://github.com/dfguan/purge_dups
sanger-tol/genomenote	v1.0	https://github.com/sanger-tol/genomenote
sanger-tol/readmapping	1.1.0	https://github.com/sanger-tol/readmapping/tree/1.1.0
YaHS	1.2a	https://github.com/c-zhou/yahs

### Wellcome Sanger Institute – Legal and Governance

The materials that have contributed to this genome note have been supplied by a Darwin Tree of Life Partner. The submission of materials by a Darwin Tree of Life Partner is subject to the **‘Darwin Tree of Life Project Sampling Code of Practice’**, which can be found in full on the Darwin Tree of Life website here. By agreeing with and signing up to the Sampling Code of Practice, the Darwin Tree of Life Partner agrees they will meet the legal and ethical requirements and standards set out within this document in respect of all samples acquired for, and supplied to, the Darwin Tree of Life Project.

Further, the Wellcome Sanger Institute employs a process whereby due diligence is carried out proportionate to the nature of the materials themselves, and the circumstances under which they have been/are to be collected and provided for use. The purpose of this is to address and mitigate any potential legal and/or ethical implications of receipt and use of the materials as part of the research project, and to ensure that in doing so we align with best practice wherever possible. The overarching areas of consideration are:

•   Ethical review of provenance and sourcing of the material

•   Legality of collection, transfer and use (national and international)

Each transfer of samples is further undertaken according to a Research Collaboration Agreement or Material Transfer Agreement entered into by the Darwin Tree of Life Partner, Genome Research Limited (operating as the Wellcome Sanger Institute), and in some circumstances other Darwin Tree of Life collaborators.

## Data Availability

European Nucleotide Archive: *Cheilosia variabilis.* Accession number PRJEB59374; https://identifiers.org/ena.embl/PRJEB59374. ( Wellcome Sanger Institute, 2023) The genome sequence is released openly for reuse. The *Cheilosia variabilis* genome sequencing initiative is part of the Darwin Tree of Life (DToL) project. All raw sequence data and the assembly have been deposited in INSDC databases. The genome will be annotated using available RNA-Seq data and presented through the Ensembl pipeline at the European Bioinformatics Institute. Raw data and assembly accession identifiers are reported in Table 1.

## Author information

Members of the University of Oxford and Wytham Woods Genome Acquisition Lab are listed here: https://doi.org/10.5281/zenodo.4789928.

Members of the Natural History Museum Genome Acquisition Lab are listed here: https://doi.org/10.5281/zenodo.4790042.

Members of the Darwin Tree of Life Barcoding collective are listed here: https://doi.org/10.5281/zenodo.4893703.

Members of the Wellcome Sanger Institute Tree of Life programme are listed here: https://doi.org/10.5281/zenodo.4783585.

Members of Wellcome Sanger Institute Scientific Operations: DNA Pipelines collective are listed here: https://doi.org/10.5281/zenodo.4790455.

Members of the Tree of Life Core Informatics collective are listed here: https://doi.org/10.5281/zenodo.5013541.

Members of the Darwin Tree of Life Consortium are listed here: https://doi.org/10.5281/zenodo.4783558.
