# Supplementary material for: The genome sequence of the Figwort Cheilosia, Cheilosia variabilis (Panzer, 1798)
Source: Wellcome Open Res. 2023 Aug 30;8:377. [Version 1] doi: 10.12688/wellcomeopenres.19851.1 (PMC10654491; doi:10.12688/wellcomeopenres.19851.1)
Supplement: Peer review history

# Reviewer report: Reviewer response for version 1

**DOI:** 10.21956/wellcomeopenres.21984.r69734

**Published:** 2023-11-16

**Authors:** Omar Rota-Stabelli

**Affiliations:** 1 Research and Innovation Centre, Edmund Mach Foundation Research and Innovation Centre, San Michele all'Adige, Trentino-South Tyrol, Italy

This data-note is ok: the genome data is great, clearly presented, the reading is smooth. So my "review" is necessarily positive.

 I noticed however that figures are quite "raw". Indeed, I inspected other data-notes from the same journal and I noticed that it is basically the same type of figures likely auto-generated by the pipeline. Therefore this type of note is just an exercise of writing 20 lines of introduction on an animal and copy and paste the pipeline output figures. Probably the most complicate part of the work is to reshuffle method's text in order to look like it is original and not a copy of previous (basically identical) text. I guess that the whole process can be automatically made by an AI. Fact is that another AI should review it, not a scientist.

 All the best.

Are sufficient details of methods and materials provided to allow replication by others?

Yes

Is the rationale for creating the dataset(s) clearly described?

Yes

Are the datasets clearly presented in a useable and accessible format?

Yes

Are the protocols appropriate and is the work technically sound?

Yes

Reviewer Expertise:

Phylogeny, phylogenomics, comparative genomics.

I confirm that I have read this submission and believe that I have an appropriate level of expertise to confirm that it is of an acceptable scientific standard.

# Reviewer report: Reviewer response for version 1

**DOI:** 10.21956/wellcomeopenres.21984.r66368

**Published:** 2023-09-21

**Authors:** Roberto Feuda

**Affiliations:** 1 Department of Genetics and Genome Biology, University of Leicester, Leicester, England, UK

This data note presents the genome assembly of Figwort Cheilosia, *Cheilosia variabilis*, as part of the Darwin Tree of Life Project. The method section is both comprehensive and clear. The genome's quality stands at a high standard, thanks to the use of HiFi and Hi-C, which generated a chromosome-scale assembly. I have a few minor suggestions to enhance this note:

1. To provide better context, it could be beneficial to elaborate on the reason for sequencing this species. For instance, how the genome will contribute to a deeper understanding of the ecological flexibility of this species.
2. In the interest of reproducibility, it is advisable to create a GitHub (or similar) page containing the commands used and the entire pipeline used for genome assembly. This will facilitate transparency and accessibility for future researchers. 

Are sufficient details of methods and materials provided to allow replication by others?

Yes

Is the rationale for creating the dataset(s) clearly described?

Yes

Are the datasets clearly presented in a useable and accessible format?

Partly

Are the protocols appropriate and is the work technically sound?

Yes

Reviewer Expertise:

Genomics, phylogenomics

I confirm that I have read this submission and believe that I have an appropriate level of expertise to confirm that it is of an acceptable scientific standard.
